# Graph Analysis of the Visual Cortical Network during Naturalistic Movie Viewing Reveals Increased Integration and Decreased Segregation Following Mild TBI

**DOI:** 10.3390/vision8020033

**Published:** 2024-05-10

**Authors:** Tatiana Ruiz, Shael Brown, Reza Farivar

**Affiliations:** 1 Department of Ophthalmology & Visual Sciences, McGill University, Montreal, QC H4A 0A4, Canadashael.brown@mail.mcgill.ca (S.B.); 2 Research Institute of the McGill University Health Center, Montreal, QC H3G 1A4, Canada

**Keywords:** fMRI, cortical networks, traumatic brain injury, concussions, graph theory, movie viewing, naturalistic stimulation, visual perception, efficiency, integration

## Abstract

Traditional neuroimaging methods have identified alterations in brain activity patterns following mild traumatic brain injury (mTBI), particularly during rest, complex tasks, and normal vision. However, studies using graph theory to examine brain network changes in mTBI have produced varied results, influenced by the specific networks and task demands analyzed. In our study, we employed functional MRI to observe 17 mTBI patients and 54 healthy individuals as they viewed a simple, non-narrative underwater film, simulating everyday visual tasks. This approach revealed significant mTBI-related changes in network connectivity, efficiency, and organization. Specifically, the mTBI group exhibited higher overall connectivity and local network specialization, suggesting enhanced information integration without overwhelming the brain’s processing capabilities. Conversely, these patients showed reduced network segregation, indicating a less compartmentalized brain function compared to healthy controls. These patterns were consistent across various visual cortex subnetworks, except in primary visual areas. Our findings highlight the potential of using naturalistic stimuli in graph-based neuroimaging to understand brain network alterations in mTBI and possibly other conditions affecting brain integration.

## 1. Introduction

Mild traumatic brain injury—a disorder with a massive incidence of approximately 56 million annually [1] induces long-lasting and debilitating cognitive deficits that are hard to explain neurometrically in humans. Since the first functional magnetic resonance imaging (fMRI) study on mTBI [2], conventional approaches using General Linear Models (GLM) have been tremendously successful in identifying regions that may be implicated in the cognitive deficits associated with mTBI [3]. A sizeable list of such cortical regions includes prefrontal [4,5], medial, and temporal [6], and the anterior cingulate functional connectivity of the anterior cingulate cortex in Veterans with mild traumatic brain injury [7] cortices. Altered functional connectivity in the visual network was correlated with visuo-spatial and cognitive dysfunction in a mild TBI group [8].

The emergent model is that the multiple regions implicated likely form multiple networks—interconnected regions that share information and depend on one another. Whereas detecting activity differences with GLM can be straightforward, detecting network changes is quite complex because networks can change by adding/subtracting nodes or connections and by adjusting the magnitude of node response. These alterations combine to essentially modify the topology of the network. Thus, making inferences about network changes requires a mathematically rigorous framework that captures network shape changes, which is found in graph theory [9,10].

Network graphs are inferred from node-based functional connectivity matrices. After traditional processing of the BOLD signal, the classic functional connectivity matrix (correlation of timeseries from each pair of voxels or voxel clusters or areas) can be translated into graph networks in one of two ways. Thresholding is applied to categorize connections between functionally significant and irrelevant processes, and correlation values can be kept reflecting connection strength (weighted graph) or simply discarded (binary graph). Parameters describing such networks, thus, fully depend on the brain state activating that network. Graph network analysis provides key insight into whether new connections are formed and whether they are random, which allows for comparison between clinical and healthy populations.

Four commonly reported parameters of network restructuring are (1) network connectivity degree (the average number of significantly correlated connections per node), (2) efficiency (the inverse of the shortest path between two nodes), (3) modularity (connections pertaining to a functional module in a case-network compared to the total number of edges in the graph), and (4) clustering (the abundance of interconnected node trios) [11]. These parameters are biologically meaningful as they quantify synchrony of processing across the cortical network (connectivity degree), the availability of local information, the tightness of connectivity in trade-off with redundancy (efficiency), the functional segregation in the architecture of a cortical network (related to modularity; see [12] for a review), and the level of local cohesiveness (clustering; see [13]). Their interpretation, thus, includes integration and segregation of processing on top of simple connection density when taken together.

These four parameters have been valuable in understanding functional network restructuring and have been related to abnormal cognitive function that TBI patients experience [14,15]. To cite only a few examples, the connectivity degree was correlated with motor–cognitive contralateral disruptions in TBI and not in healthy controls [9]. Raizman et al. (2020) [16] reported a correlation between efficiency and nonverbal abstract reasoning in a group of healthy controls but not in the TBI group. On the other hand, cognitive training was found to reorganize network modularity in TBI [17], and training was predictive of modularity in another study [18]. In a resting state study, mild TBI was associated with increased clustering, which was negatively correlated with post-concussive symptoms [19].

Ideally, network descriptors should add value to our general understanding of cortical changes after mTBI and consider the dynamic aspect of cortical function. The rapid cortical responses to internal demands or external factors, however, are overlooked when network behaviour is deemed constant, for example, when network organization is inferred from resting state data [15]. While the studies above looked at network architecture in relation to the performance of cognitive tasks, others assessed baseline changes that are manifest in a resting state—when a patient lies quietly in the scanner while their brains are scanned [20]. Han, Chapman, and Krawczyk (2016) [21] analyzed the resting-state data of 40 TBI patients suffering from chronic symptoms (8 years after injury on average) using network analytic approaches and reported increased connectivity and decreased efficiency, which they took to implicate weaker integration and, thus, poorer information flow. Results from studies of acquired brain injury [22] and TBI patients of various severity [23] have been understood similarly—after acquired damage to the cortical network, integration is compromised. Thus, TBI may impair cortical integration even at rest.

Resting state connectivity can be informative of *baseline* changes potentially caused by TBI and has been analyzed using graph metrics [24,25,26] while classic cognitive tasks pose problems for ecological validity. This is especially true in the context of mTBI. The healthy brain can be comprehended as fluctuating between intense functional states demanded by difficult tasks and resting state [20]. However, we have no certitude that injury affects these brain states equally or even proportionately. It is likely that mTBI causes the cortical system to behave as if it were constantly under load because patients experience cognitive fatigue from completing usual tasks [27]. Patients struggle with cognitive fatigue in the absence of excessive demands, so results from demanding tasks might place them in an uncharacteristic state of functional activity [28] or misrepresent their cognitive function because they could be at floor performance.

This presents us with a conundrum, whereby we cannot fully infer mTBI dysfunction from resting-state changes (because they could be well compensated during natural activity), and we cannot achieve this from targeted tasks (because they may represent “lab” behaviour and are often more difficult than real-world tasks). To overcome this conundrum and carry out a network analysis of mTBI changes during an ecologically valid task, we opted for visually complex, narrative-free movie scenes to achieve a level of engagement consistent with familiar, everyday activities.

We predict the mTBI group to exhibit a higher connectivity degree than the healthy controls as this had already been demonstrated during fMRI and attributed to compensation [29,30]. Efficiency is predicted to be higher in the mTBI group if the increased degree is not random but caused by purposeful compensatory mechanisms instead [17]. This parameter reflects the integrative capabilities of the system and describes how well information travels from one cortical area to another. In contrast, the tightness and segregation of processing is reflected by the modularity of the graph. In the mTBI group, modularity is expected to decrease as compared to healthy controls because the boundaries between functional modules are blurred by active degeneracy, and the connections are less specialized [31]. We expect clustering to be increased in the mTBI group as a marker for strengthened local cohesiveness and specialization [32]. We, thus, expected stronger connections between modules and better long-range integration combined with short-range specialization, all while actively being engaged in a natural viewing task.

## 2. Materials and Methods

### 2.1. Participants

All participants gave their informed consent prior to taking part in the experiment. All procedures were in accordance with the Code of Ethics of the World Medical Association (Declaration of Helsinki) and were approved by the Research Ethics Board of the McGill University Health Center (Montreal, QC, Canada).

All participants were screened for anomalous vision loss or vision disorders (glaucoma, retinal detachment, macular degeneration, etc.). They had normal or corrected to normal visual acuity (wore their usual refractive correction if lenses). The average age of the participants was 36 years old (*SD* = 10. years, *n* = 17) in the mTBI group and 26 years old (*SD* = 6 years, *n* = 54) in the control group.

#### TBI and Control Participants

Participants were recruited through the McGill University Health Center out-patient TBI clinic. The diagnostic criteria for mild TBI were Glasgow Coma Scale score between 13 and 15, less than 30 min of loss of consciousness, and less than 24 h of amnesia regarding events immediately before or after the accident. Patients with mild TBI who gave their authorization to be contacted went through a phone screening interview. The exclusion criteria were (1) family history of epilepsy or seizure or the administration of prescription medication with increased risk of seizure, (2) severe tremors or involuntary movements, (3) general anesthesia in the past 6 months, (4) mTBI occurred less than 1 month ago or more than 2 years ago, (5) a history of multiple brain injury. Following our previous publication, participants filled out a questionnaire adapted from Assessment with Mild Traumatic Brain Injury for the Defense Centers of Excellence for Psychological Health and Traumatic Brain Injury [33], investigating blurred vision, migraines, behavioral change to palliate visual discomfort, etc. None of our self-reported and neuropsychological measures (clock drawing test, trail making test, bells test) correlated with any of our neuroimaging results. The final sample size of tested mTBI participants was 17 (9 females).

Healthy participants were recruited through public announcements in the Montreal General Hospital and on social media. Exclusion criteria included conditions 1–4 outlined above and no history of any acquired brain injury. The control group was comprised of 54 individuals (28 females).

### 2.2. Stimuli and Procedure

Stimuli were presented using MATLAB^®^ (2014b, The Math Works Inc., Natick, MA, USA) and synchronised with acquisition start time by Sterescopic Player (http://www.3dtv.at; accessed on 1 August 2013) and ActiveX connection using a 10-bit graphics card (Nvidia Quadro 2000) on a gamma-calibrated 3-D LCD BOLD screen reflected by a mirror above the participants’ head. They were placed at a 170 cm viewing distance from the monitor, spanning 9.4 by 17 degrees of visual angle at a pixel resolution of 1920 by 1080. Participants were scanned while watching two five-minute movie clips twice, once in 2D and once in 3D (using polarized glasses), cut from the movie “Under the Sea 3-D: IMAX” [34]. Whether participants saw two of the four clips in 3D was verified after each scanning session. Scenes included marine fauna and flora, constituting naturalistic stimuli with no human-made object or other element that could have biased representation depending on culture, gender, or age. Participants were instructed to fixate on the center of the screen (white fixation cross present for the entirety of the stimuli), and a blank screen with fixation cross was presented before each clip for four seconds.

**Figure 1:**
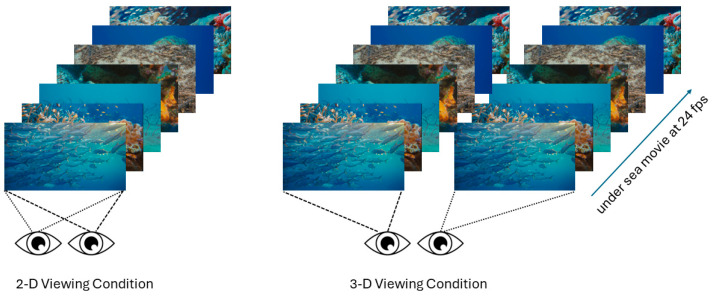
The two viewing conditions and frame grabs of the movie clip (IMAX underwater documentary). In the monoscopic viewing condition (**left**), only one of the two viewpoints was shown, while in the stereoscopic viewing condition (**right**), both views were seen and fused by the subject.

The stimulus and viewing conditions are depicted below in Figure 1. 

#### Data Acquisition

FMRI data were acquired on a 3T Siemens TIM Trio scanner (TR = 2000 ms, Resolution 3 mm^3^, TE = 30 ms, flip angle = 76, matrix size = 64 × 64, Field of View = 192 × 192 mm, number of slices = 37, interleaved acquisition, R = 2, GRAPPA acceleration, coronal orientation, head-foot phase encoding) at the Montreal Neurological Institute (McGill University Health Center) using the posterior 20 channels of the Siemens 32ch head coil, thus capturing the posterior of the head, with an approximate cut off near the central sulcus. Initial 3 EPI scans were discarded for T1 stabilization, and only the remaining 120 volumes were retained for further analysis. Anatomical data (T1-weighted multi-echo magnetization prepared—rapid gradient echo sequence—MEMPRAGE—1 mm isotropic resolution) was acquired with the full 3d channel coil after the functional imaging. In addition, we acquired a set of whole-head EPIs in both head-foot and foot-head encoding for follow-up distortion correction and for registration to anatomical images.

### 2.3. Data Processing

**Figure 2:**
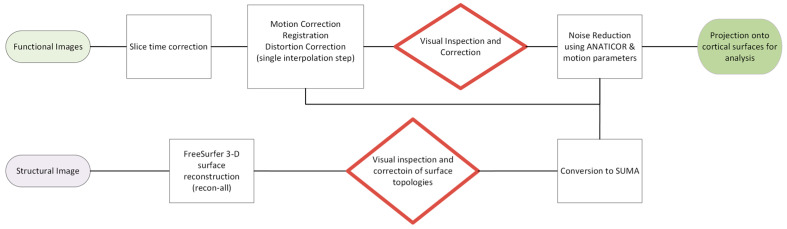
Data processing pipeline. Note that all spatial transformations were carried out in a single step, and the results of the pre-processing steps were visually inspected for validity prior to next steps.

All data processing are depicted in Figure 2 below. 

#### 2.3.1. Preprocessing

fMRI data were preprocessed with Analysis of Functional NeuroImages (AFNI) [35]. Slice time correction was carried out using 3dTshift in the AFNI toolkit, with default settings (referenced with respect to the middle slice, Fourier interpolation). To minimize spatial blurring, we applied all spatial transformations in a single step following slice-time correction. Motion was determined using 3dvolreg with reference to the motion-corrected average of the first run, and the transformation matrices were stored. Subsequently, distortion was estimated using the up–down method as implemented in the 3dQwarp and estimated from the whole-head EPI images that were registered to the average motion-corrected volume of the first run; again, the distortion map was then stored for subsequent concatenation with other spatial transformations. Finally, the whole-head undistorted volume was also registered to the anatomical images using the align_epi_anat.py script from AFNI using mutual information as a cost function. Once all spatial transformations were estimated, they were concatenated and applied at once using 3dNwarpApply with quintic interpolation. We also applied detrending and denoising (to remove structured noise along white matter boundaries from the time series) algorithms using ANATICOR [36] and also included the square and the derivative of the motion parameters in the detrending/denoising step with ANATICOR.

#### 2.3.2. Surface-Based Analysis

After pre-processing, all data were projected onto cortical surface meshes for the group analysis. Cortical surfaces were first extracted for each subject using their T1-weighted image, using the Freesurfer package (http://surfer.nmr.mgh.harvard.edu/; accessed on 1 September 2018), and corrected errors after visual inspection. The Freesurfer surfaces were then converted to SUMA [37] using a standard mesh model with 32,000 nodes (ld40; [38]). We selected a mesh of 32,000 nodes (per hemisphere) to maximize resolution and optimize graph computation power. Statistical analysis was performed on surface-projected data because they preserve individual subjects’ topology and allow for better domain-matching across subjects, which strengthens statistical power compared to voxel-based analysis [39]. In this scheme, each node from one subject corresponded to the same node from other subjects, which allows for inter-subject comparisons.

#### 2.3.3. Graph Comparisons

To find global and local differences in functional connectivity (FC) between groups, we computed multiple measures of network topologies on thresholded FC in mTBI participants and healthy controls. These measures were chosen because they quantify, in terms of network structure, the notions of functional integration and segregation—both of which we expected to differ between the mTBI and control groups.

Functional connectivity matrices were calculated as correlation matrices (product-moment correlation) between pairs of timeseries for each of the 32,000 nodes for each subject and each movie clip. We then thresholded the 32 k × 32 k correlation matrix using false-discovery rate (FDR) correction controlled at q* = 0.001 [40]. On average, 5 to 10% of correlations were maintained. Correlation matrices were, thus, converted into cortical network graphs.

Thresholded FC matrices induce an undirected, unweighted network structure. An undirected, unweighted network (graph) is a set of vertices $V=\left(v_{1},v_{2},\ldots ,v_{n}\right)$ and edge connections between pairs of vertices $E=\left(e_{1},e_{2},\ldots ,e_{k}\right)$. In our example, the nodes in V will represent a node in the cortical mesh models, and edges E represent pairs of cortical nodes whose functional correlation values survived the thresholding.

**Figure 3:**
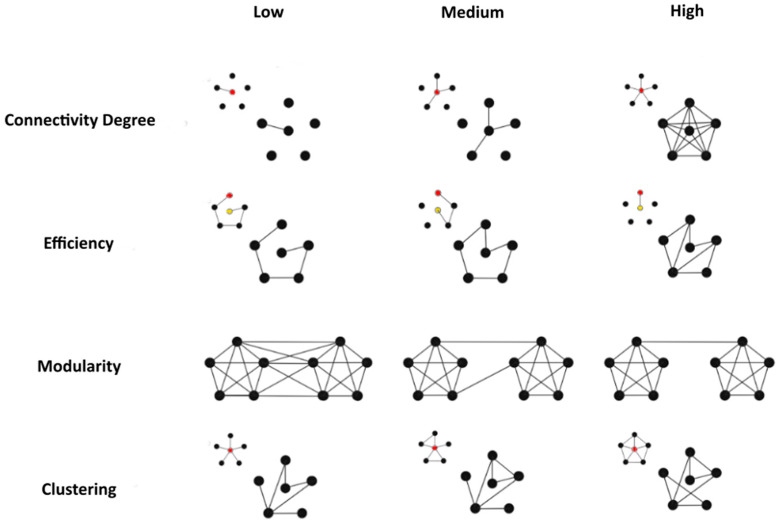
Measures of network organization. Schematic illustration of increasing levels of connectivity degree, efficiency, and modularity. Nodes are represented as dots, and edges are represented as lines connecting the nodes. While the measures are estimated over the whole network, they are calculated either for every node or for all node pairs. Average connectivity degree represents the average number of connections a node may have—for a single node (i.e., the red node), connectivity degree means the number of nodes it is connected to. Average efficiency represents degree of connectivity between pairs—moving from one node (red) to another (yellow) involves many intervening points in the “low” efficiency level, and the number of intervening points decreases as efficiency in the network increases. Modularity captures the extent to which nodes cluster together and away from other clusters. Clustering captures the extent to which all possible connections between nodes are realized—the graph with lower clustering has fewer connections realized compared to the network with higher clustering.

We hypothesized that TBIs have reduced segregation and increased integration due to compensation by their unaffected pathways and circuitry. To test this hypothesis, we computed four measures of how well information is communicated globally and locally in graphs: (1) mean degree of nodes, (2) global efficiency, (3) modularity, and (4) clustering. These were calculated in R [41]. For a graphic illustration of the network measures explored, see Figure 3.

The degree of a node $v_{i}$, $d\left(v_{i}\right)$, is the number of edges connected to $v_{i}$. As such, the mean degree of the whole network *D*(*G*) is the mean of all nodal degrees and measures average local connectivity across the whole graph (see illustration of connectivity concepts in Figure 3).

The global efficiency of a graph *G* was measured as the average of reciprocal distances—the minimum number of edges needed to walk between two nodes, denoted by $d_{i,j}$—between all pairs of distinct nodes $v_{i},v_{j}$ where *i* ≠ *j*: $E_{global}\left(G\right)=\frac{1}{n\left(n-1\right)}\sum_{i\neq j}\frac{1}{d_{i,j}}$ [42]. The fraction before the summation accounts for the number of pairs of vertices in the graph. Intuitively, $E_{global}\left(G\right)$ is large when many distances are small, which is when most nodes are separated by short walks in the graph, which, in effect, is a measure of integration in the network.

Given a parcellation of nodes into functional areas (i.e., assigning a functionally relevant [43] label such as “primary visual cortex”, V2, V3, etc., to each node in the graph), we can ask how modular the network is with regard to that parcellation. Intuitively, modularity relates to how specialized/segregated different regions of the brain (graph) are in processing information. We can quantify this notion in the graph by $Q\left(G\right)=\frac{1}{2k}\sum_{i,j=1}^{n}\left[A_{i,j}-\frac{d\left(v_{i}\right)\cdot d\left(v_{j}\right)}{2k}\right]\cdot \delta \left(r_{i},r_{j}\right)$, where $A_{i,j}$ is the binary variable representing whether there is a connection between vertices $v_{i}$ and $v_{j}$, $r_{i}$ is the region label of vertex $v_{i}$ and $\delta \left(r_{i},r_{j}\right)$ is 1 if its two inputs are the same and is 0 otherwise [44]. In essence, modularity grows as the number of edges within a defined region grows.

Finally, the clustering coefficient can be understood as a resiliency marker for a given network. When two nodes are connected to each other, whether they are both connected to the same third node or not determines the local stability of processing. Information has more paths on which it can travel between its source and destination on the local scale when edges form triangles between three nodes. The clustering coefficient $C_{i}$ of a node $v_{i}$ is the proportion of completed triangles, i.e., of $A_{j,h}=1$ when $A_{i,j}=A_{i,h}=1$, defined as $C_{i}=\frac{1}{d_{i}\left(d_{i}-1\right)}\sum_{j\neq h}A_{i,j}\cdot A_{i,h}\cdot A_{j,h}$ [45]. The clustering of a graph is simply the average of all node-specific clustering coefficients.

#### 2.3.4. Whole-Brain Networks and Regional Subnetworks

We also sought to study graph structures in subnetworks in addition to whole-brain networks (as above). For subnetwork analyses, we divided the cortex into early, dorsal, ventral, and fronto-parietal regions as defined by a large-scale atlas [43]. We defined a subnetwork as a subset of the vertices of the graph, *V*′⊆*V*, and the edges of the subnetwork are the edges of the full graph, which connect vertices in *V*′. Because modularity is not meaningful within a subnetwork, we only calculated mean degree, efficiency, and clustering of the subnetwork graph of each functional region in the parcellation. All graph analytic estimates were carried out using the igraph (version 1.2.5) and brainGraph (version 2.7.3) packages in the R (version 3.6.3) programming language.

To estimate the effects of mTBI on the various network measures outlined above, we computed a mixed model factorial ANOVA with Group (mTBI vs. Controls) × Movie (3D vs. 2D) for each measure of network organization (mean degree, efficiency, and modularity).

For stream subgraphs and region subgraphs, there was a separate model for each of the early, dorsal, ventral, and fronto-parietal subnetworks and regions, respectively. The significance and sign (positive or negative) of the *β*_*tbi*_ were the focus of our results; however, the interaction terms, including TBI effects, were also examined.

For thorough introductions to graph theory for neuroscience, see [11,14].

## 3. Results

**Figure 4:**
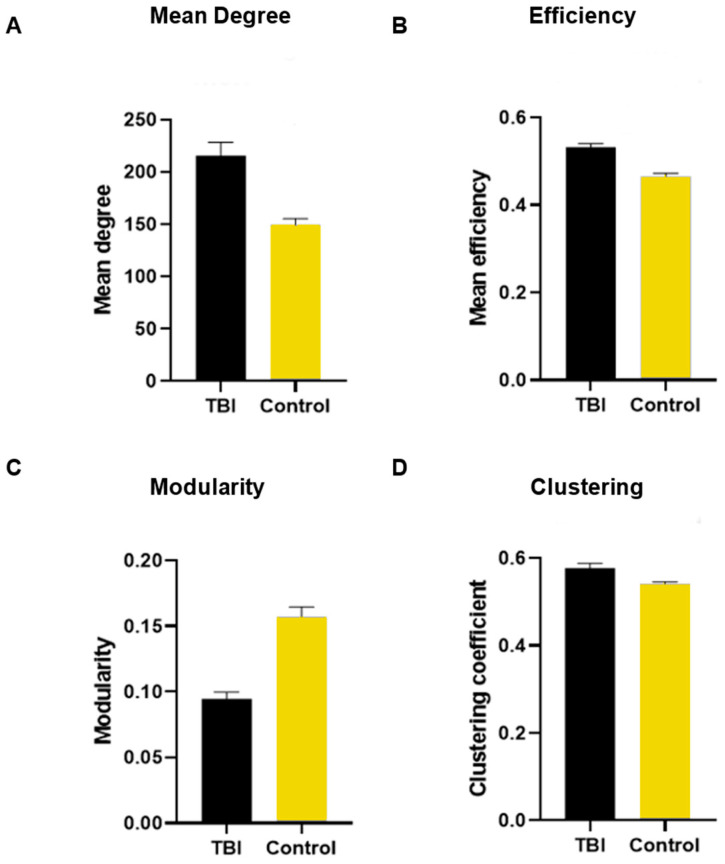
Global network measures of the visual cortex during naturalistic viewing in mTBI participants and healthy controls. All measures reached statistically significant differences between the two groups.

We first consider the linear effects of traumatic brain injury on (1) mean degree, (2) efficiency, (3) modularity, and (4) clustering coefficient in thresholded FC graphs in subnetworks or the full visual cortex (Figure 4). In all models, there were no meaningful interactions, suggesting that the stimulus (2D/3D) did not alter the effect of mTBI on FC architecture. However, the stimulus condition had a main effect on connectivity degree: 3D yielded higher connectivity than 2D (*β* = 24.4, *p* < 0.05). We related all our findings with our visual symptom questionnaire and found no correlation between each question and no correlation with the total score.

### 3.1. No Change in Static Architecture of Natural Viewing Network after mTBI

There was no major restructuring of the visual cortex network engaged in natural movie viewing between groups—the regions of interest engaged in the control group were the same in the mTBI group and they were connected in a similar network. The dynamic aspect of these connections, however—the degree, efficiency, modularity, and clustering ruling their interaction—was altered.

In the full-graph models (all nodes from the visual cortex), mTBI had significant effects on all metrics: mean degree (*β* = 88.4, *p* = 1.30 × 10^−6^) (Figure 4A), efficiency (*β* = 0.08, *p* = 4.28 × 10^−5^) (Figure 4B), modularity (*β* = −0.07, *p* = 0.0002) (Figure 4C), and clustering (*β* = 0.04, *p* = 0.001) (Figure 4D).

The increased mean degree and efficiency support the hypothesis of overcompensation in mTBI, however, in different ways. An increase in mean degree reflects a global increase in connectivity, which agrees with known results of increased functional connectivity in mTBI subjects, whereas an increase in graph efficiency points to greater structured reorganization of key connections in the visual cortex [46].

**Figure 5:**
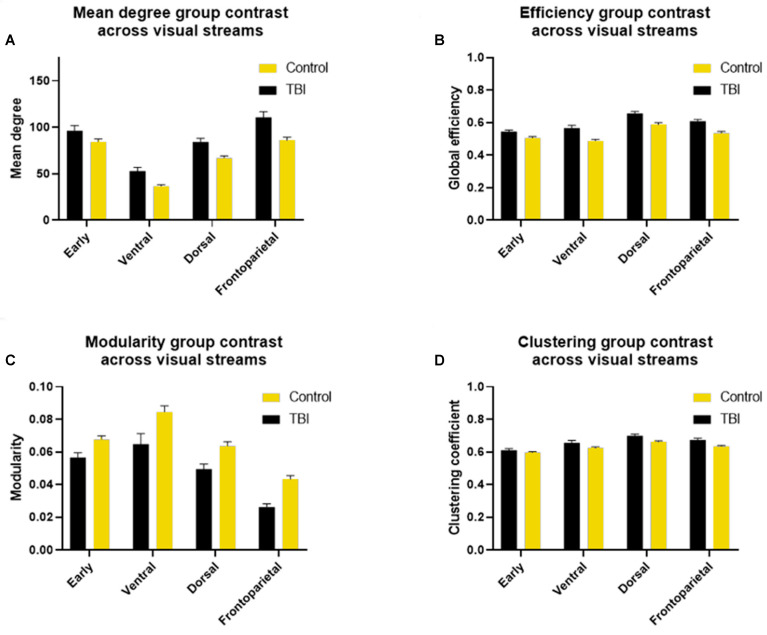
Measures of subnetwork organization during naturalistic viewing in mTBI and healthy control participants.

In the subnetworks (Figure 5), the mean degree was altered in the mTBI group in the ventral subnetwork (*β* = 21, $p_{adjusted}$ = 0.0005), in the dorsal subnetwork (*β* = 23, $p_{adjusted}$ = 0.004), and in the fronto-parietal subnetwork (*β* = 38, $p_{adjusted}$ = 0.002) (Figure 5A). Efficiency was also significantly impacted by mTBI in the ventral subnetwork (*β* = 0.1, $p_{adjusted}$ = 0.004), in the dorsal subnetwork (*β* = 0.08, $p_{adjusted}$ = 0.01), and in the fronto-parietal subnetwork (*β* = 0.09, $p_{adjusted}$ = 0.005) (Figure 5B). The combined increase in mean degree and efficiency in the ventral, dorsal, and fronto-parietal but not in the early visual areas provides evidence that these particular subnetworks drive the global structured alteration following mTBI reported above. If the mean degree was increased alone, and efficiency was not different, the increase in connectivity would have been diffuse and unstructured. Interestingly, efficiency was increased evenly across subnetworks, but the mean degree showed a two-fold stronger increase in the fronto-parietal subnetwork.

Modularity was decreased only in the fronto-parietal stream (*β* = −0.02, $p_{adjusted}$ = 0.004). We expected that any effect of TBI on modularity would be a negative one, reflecting decreased segregation of processing, and that was confirmed in our analysis (Figure 5C).

Clustering was increased in the dorsal subnetwork (*β* = 0.05, $p_{adjusted}$ = 0.02) and in the fronto-parietal subnetwork (*β* = 0.06, $p_{adjusted}$ = 0.004) (Figure 5D).

### 3.2. Increased Efficiency in Specific Regions of Interest in the mTBI Group

The within-region analysis only looked for effects on efficiency in subgraphs, and the regions which showed a strong effect of TBI were VO2 (*β* = 0.12, $p_{adjusted}$ = 0.004), PHC (*β* = 0.09, $p_{adjusted}$ = 0.008), V3a (*β* = 0.08, $p_{adjusted}$ = 0.03), IPS0 (*β* = 0.09, $p_{adjusted}$ = 0.02), IPS1-2 (*β* = 0.08, $p_{adjusted}$ = 0.05). All other visual areas did not show any difference in efficiency between the two groups.

## 4. Discussion

For the first time, we have shown that natural movie viewing is a powerful paradigm for revealing network changes in mTBI with ecologically valid levels of demand on the patient, and with this paradigm, we provided evidence for the idea that injury alters the cortical networks. Overall, our results point towards two strategies of connectivity change in mTBI patients powered by an increase in connectivity. The increase in integration (efficiency and mean degree) is counterbalanced by a decrease in segregation (modularity) at the macro scale, but at the micro scale, specialization is increased (clustering).

### 4.1. Higher Mean Connectivity Degree

First, we found a significant global increase in connectivity throughout the visual cortex (whole graph) in the mTBI group. This finding was repeated specifically in regions of the visual cortex dedicated to complex processing of information—cortical regions beyond V4 in the visual system hierarchy, pertaining to both the ventral and the dorsal streams as well as the fronto-parietal region. The mean connectivity degree, when calculated from binary graphs, can be understood as equivalent to the significance of the functional connectivity analysis. Our results, thus, corroborate previous evidence of increased functional connectivity after mTBI [30,47]. An increase in functional connectivity, or in mean connectivity degree, does not inform us about the changes to the structure of cortical networks after injury. For this reason, we compared other measures of network organization between healthy controls and mTBI participants as an investigation of network reorganization following mTBI.

### 4.2. Higher Global Efficiency

Second, we found a significant increase in global efficiency throughout the visual cortex, and within regions dedicated to complex processing of visual information (beyond V4) as well. In the global graph of the visual cortex and within the ventral, dorsal, and fronto-parietal subgraphs, nodes were generally less functionally distant to other nodes in the mTBI group than they were in healthy controls—there were fewer edge bridges to take between two nodes. Another way to appreciate increased efficiency is to understand it as greater integration in the mTBI group. This finding needs to be interpreted in parallel with the first one because they complement one another—the increase in connectivity degree could have been diffuse if not for the increase in efficiency and clustering without an increase in interregional connections—and this is discussed further below. Interestingly, both parameters were increased both in the whole visual system network but also in the same specific subnetworks. Together, these two findings show a purposeful increase in connectivity towards better global integration, which suggests compensatory mechanisms are involved in network reorganization following mTBI.

### 4.3. Lower Modularity

Our third finding was that of a decreased modularity after mTBI in the whole visual cortical network as well as within the fronto-parietal subnetwork. This subnetwork is particularly important for the integration of complex visual features (e.g., [48]) that occur in scenes in motion. A decrease in modularity in this subnetwork suggests higher co-recruitment of modules to process feature integration in the mTBI group. Decreased modularity combined with an increase in efficiency reflects an increase in functional segregation. These two parameters’ changes overlapped in the complete visual cortex network and in the fronto-parietal subnetwork, suggesting that segregation is affected by mTBI, specifically at high levels of processing. We should note that based solely on the connectivity results, we would have expected an increase in modularity. The fact that modularity was decreased in the mTBI group is a strong indicator that the network changes were not diffuse and the change had a functional purpose.

### 4.4. Higher Clustering

We found increased clustering in the mTBI group in the whole visual network and within the dorsal and fronto-parietal subnetworks. Thus, these two subnetworks drove the global results, and there are specific areas where there were more interconnected triplets of nodes in the mTBI group than in healthy controls. Our results are in line with previous publications using the graph analysis of neuroimaging data from mTBI patients [49,50,51] and moderate to severe TBI patients [52]. A high clustering coefficient reflects a network with many cliques of nodes. The strategic accumulation of clusters increases segregation for local specialization [32]. Functional specialization at the local scale was increased. Higher clustering might be in line with an adaptative or compensatory reorganization of the visual network following mTBI; however, it can be a costly one—it supposes an increase in the number of steps needed to go from one node to another [46]. This is corroborated by findings of increased path length following mTBI [32] but it is not in contradiction with the increase in efficiency that reflects a decrease in steps needed to join two nodes at a global scale. Two distant nodes tended to be functionally closer in the mTBI group than in healthy controls, but two closely linked nodes tended to be functionally farther away in the mTBI group.

Taken together, these findings reflect a functionally relevant reorganization in the topology of long-range connections, which facilitates inter-regional communication for the complex processing of visual information. It suggests as well that regions functionally defined via probabilistic methods in healthy controls might be working together following mTBI to solve processing problems usually handled by single modules in healthy controls. Finally, the increase in local specialization contrasts with the decrease in global specialization, and these effects overlap locally only in the fronto-parietal subnetwork.

### 4.5. Graph Theory and mTBI

Previous studies using resting-state fMRI reported increases [30,53] but also decreases [29,54] in functional connectivity following mTBI at rest, and these changes were both paradoxically correlated with behavioral performance and mTBI symptomatology [19,55]. The present paper shows that connectivity degree is increased in the cortical networks engaged by natural movie viewing as well, although we could not relate our findings to visual complaints. We speculate that the decrease in activity in the early visual cortex [56] and the compensatory increase in mean connectivity degree throughout visual areas are maintained across very different brain states provoked by very different processing demands because the injured brain is constantly overloaded.

We are aware that our results regarding global efficiency are not in line with some of the previous literature on mTBI [19,57]. However, we believe that our choice of task and stimuli (instead of using resting state) drove this difference—the increase in connectivity measured during resting-state fMRI could be unstructured and reflective of diffuse damage to the global cortical network, whereas natural viewing could recruit one or a few specific networks with tight processing within their nodes. In this scenario, natural stimuli could represent an active task that elicits efficient visual processing while the resting-state reflects spontaneous functional networks, meaning participants are not engaged in a common task that would grasp any particular network.

The idea that the brain is stuck in a cognitive state or experiences difficulties switching between one state and another has been previously discussed but has yet to be related to network measures. For example, concussed adolescents were found to be “stuck” in one of the three cognitive states investigated in a pilot study by Muller and Virji-Babul (2018) [47], with the “stuck” state being one that specifically recruited attentional networks. From the perspective of network control theory, the architecture of a network constraint which transitions is easy to execute, and these states are easy to maintain [57,58] Mathematically, greater modularity makes it easier to transition between states and to keep a given state [58]. Much like a pure conceptual network in physics, the human brain can be conceived as a complex system which can be manipulated into a cognitive state by changing excitatory and inhibitory input it receives as “cognitive control” [59]. This could mean that the less modular a cortical network becomes following an mTBI, the more difficulties experienced by the patient in terms of state switching and maintaining a state (i.e., for sustained attention). Although we did not find a relationship between visual complaints and modularity, others have shown that cognitive training was limited by decreased modularity [18].

Recent studies investigating the balance in connectivity within and between networks following mTBI have not reached a consensus, possibly because functional segregation and integration might be task and network-dependent. Using resting-state fMRI for a connectome-wide investigation of the cortical network following TBI, connectivity was reportedly increased within networks and decreased between networks [60]. This imbalance would support the idea that injury increases segregation.

In contrast, graph analysis of cortical networks estimated from fMRI during an N-back memory task revealed decreased segregation between task-positive networks and the Default Mode Network (DMN) and increased connectivity within the DMN but not task-positive networks specifically during the more cognitively demanding condition in the mTBI group [61]. It is possible that our results denote a cognitive state between these two levels of demands. At low levels of cognitive load, another study using an easier N-back task reported no change in segregation between task-relevant networks but found increased connectivity within networks as we did, but in our case, using naturalistic viewing [62].

### 4.6. Limitations

The present paper relies on a novel application of complex network theory to analyze stimulus-driven functional connectivity and has revealed three important new findings—when watching a naturalistic movie, the visual cortex is (1) more connected, (2) more efficient, and (3) less modular after mTBI. To draw robust conclusions about whether connectivity changes following mTBI are structured or unstructured, we would like to compare our results of increased efficiency and clustering with a rewired null model—where edges are shuffled to see whether architectural descriptors were mainly driven by the increase in connectivity degree or whether the changes were structured. Although our results are based on high-resolution graphs (32,000 nodes per hemisphere), nodes cannot be understood as neurons, and edges cannot be understood as axons. Ideally, we consider the neuron to be the smallest processing unit, but it is not currently possible to evaluate them individually in humans, especially not in the context of network science. For this reason, it is noteworthy that the activity of the nodes on which functional connectivity was based and, thus, the graphs were constructed is an average of all BOLD activity in these nodes. On another level, the graphs analyzed here were binary—non-weighted—and so we have not looked at inhibitory or excitatory interactions, although these might be relevant to understanding mTBI [63,64,65,66].

## Data Availability

The data that support the findings of this study are available upon request from the corresponding author. The data are not publicly available due to privacy or ethical restrictions.
